# Combining Language Training and Work Experience for Refugees with Low-Literacy Levels: a Mixed-Methods Case Study

**DOI:** 10.1007/s12134-023-01028-6

**Published:** 2023-03-25

**Authors:** Anne Kuschel, Nina Hansen, Liesbet Heyse, Rafael P.M. Wittek

**Affiliations:** 1 Department of Social Psychology, University of Groningen, Grote Kruisstraat 2/1, 9721TS, Groningen, The Netherlands; 2 Department of Sociology, University of Groningen, Groningen, The Netherlands

**Keywords:** Refugees, Integration, Language, Work experience, Agency, Training

## Abstract

Learning the host society’s language and finding a job are important steps for the societal integration of refugees. Especially language proficiency is a key barrier for the integration of low literates. Often language training and gaining work skills are separated during the integration procedure. We investigated a 1-year pilot program for refugees with low-literacy levels in the Netherlands, which combined language training (daily classes, work-related language) with work experience in sheltered employment (second-hand shop) to facilitate language learning and prepare this group better for the labour market. Building on Ager and Strang’s conceptual integration framework (2008), we expected that this combined program should improve agency (communication strategies, preparedness for the labour market) via intergroup contact at work. We used a mixed-method approach to follow the development of participants (*N*=10) longitudinally (baseline, after 6 months, after 11 months). We gathered questionnaire data, interviewed teachers and students, and observed interactions in classes and at work. Overall, use of communication strategies increased. Analyzing individual cases (profiles) offered nuanced insights into for whom and why the program seems to have differential impact, especially in terms of labour market preparation. We discuss results and the importance of creating intergroup contact to facilitate integration in a new society.

**Supplementary Information:**

The online version contains supplementary material available at 10.1007/s12134-023-01028-6.

## Introduction

For the European Union, 2015 marked the year that more asylum applications were registered than during the last 30 years, reaching more than 1 million first-time asylum seekers (Eurostat, 2016; Konle-Seidl & Bolits, 2016). In light of this increased forced migration, many European countries face the responsibility to sustainably integrate these newcomers into society. Research indicates that the most sustainable and cost-effective strategy towards long-term integration in a host society is the participation of refugees in the labour market (“Integration in the Labour Market,” n.d.; SER, 2019). A speedy labour market integration allows refugees to become active members of their host societies and promotes well-being (Cobb et al., 2019), but also decreases dependence on welfare benefits and loss of human capital (Fasani et al., 2019). This research investigated how a combined language and work experience integration program for newcomers with low literary levels can prepare them to find work.

Psychological research indicates that both language learning and social contact are important facilitators of refugees’ integration in the host society (e.g. Tip et al., 2019). However, for many, language proficiency is a major barrier to finding a job (Gazzola, 2017; Grünhage-Monetti & Braddell, 2017). Low language proficiency also hampers the development of social contacts with natives that prevents further integration in society, including finding a job. The process of entering the workforce can take especially long for refugees with low levels of literacy (CBS, 2020; SER, 2019).

To promote earlier economic integration, governments and non-profit organizations increasingly initiate projects that aim to facilitate the language proficiency and social contacts of refugees early during the integration procedure. However, to date, evidence-based trainings for second-language (L2) learning and labour market preparation of refugees are scarce, especially for low literates. The current research investigated a pilot of 1-year combined language and work experience training program, which was initiated and implemented by a Dutch vocational training in collaboration with the municipality of Groningen in anticipation of a change to the Dutch integration law (see supplementary information). This pilot aimed to increase language use of refugees with low literacy levels and to help them improve their social contacts by acquiring work experience in a sheltered employment organization. We add to the current literature by employing a mixed-methods approach focussing on the language use and agency of the understudied group of low-literate refugees and by exploring factors that can explain differential training success. Offering a combined language and work experience program for low-literate refugees should stimulate agency through increased language use and better labour market preparation. This is especially crucial in the Dutch integration system that strongly focusses on refugees’ individual responsibilities and self-sufficiency (see supplementary information).

### Theoretical Background

#### Integration as an Interactive and Multilevel Process

Our theoretical perspective is based on the view that integration is an interactively achieved, multilevel process that includes participation and inclusion of newcomers in the host society (Ager & Strang, 2008; Strang & Ager, 2010). Labour market participation is considered a crucial element of successful integration in a host society, next to participation and inclusion in other domains such as education, housing, and access to health care. Language ability, cultural knowledge, and safe environments facilitate these outcomes. In addition, social connections between newcomers and other citizens of the host society drive integration on a local level (Ager & Strang, 2008). Based on this conceptual framework, we first present existing research on combined integration trajectories, before reviewing the interconnectedness of language and intergroup contact in more detail, as well as their relation with the development of agency.

The combined language and work experience program for low-literate refugees investigated in the current research was designed on the premise that integration is an interactive multilevel process, therefore simultaneously combining education with work experience, facilitating cultural knowledge and language learning in a safe environment, and nurturing social workplace connections. Two European taskforces (i.e. LIAM, Language for Work) previously highlighted the potential of such combined integration approaches due to the inherent interconnectedness of language and employment. As such, work can be a learning environment with opportunities for language use and social connections (Grünhage-Monetti & Braddell, 2017) while promoting the development of work skills (McHugh & Challinor, 2011). Ideally, combining language and work training facilitates the development of literacy, self-sufficiency, and early societal participation, elements essential for the integration of low-literate refugees. Indeed, a Swedish quasi-experiment indicates that the dual approach of combining language with work experience results in a quicker transition into the labour market for immigrants with weak language skills (Delander et al., 2005).

#### The Importance of Language and Intergroup Contact for Integration

In the context of refugee integration, intergroup contact is defined as contact with members of the host society (bridging social capital, Kanas et al., 2011). This intergroup contact is generally facilitated through proficiency in the dominant language and may thereby reduce social isolation (Tip et al., 2019). Indeed, language proficiency has been shown to increase the social contacts of refugees (Cheung & Phillimore, 2014).

One vital source of intergroup contact is economic integration, as work experience in the host country enables continued contact with natives. Through this intergroup contact, early work experience provides an opportunity to experience the host countries’ culture, to gain access to relevant knowledge and information (e.g. where to look for work, work-related norms, salaries), and to practice the language. This foundation facilitates access to services, wider support networks, and a broader range of labour markets (Kanas et al., 2011; Martinovic et al., 2009). Both intergroup contact and language contact can thus facilitate each other, especially so in the work context.

Furthermore, both language and intergroup contact influence refugees’ well-being and psychological integration. Refugees often lack these contacts and support networks (Marinucci & Riva, 2021; Pernice & Brook, 1996), which decreases their well-being (Holt-Lunstad et al., 2015). For example, in an Australian study on integration, refugees described weak language proficiency and communication abilities as the biggest strain on their well-being (Watkins et al., 2012). In contrast, positive contact with host country members may temper the impact of stressors such as depression or family separation (Beiser & Hou, 2001; Cobb et al., 2019; Marinucci & Riva, 2020). Intergroup contact at work may therefore foster integration.

In sum, the social dimension of integration facilitates labour market integration (European Union Agency for Fundamental Rights, 2019), and language proficiency enables employment as well as social participation (Chiswick & Miller, 2003; Lim, 2021; Stevens, 1992). Research in the Netherlands confirmed that language, work experience, and contact with natives foster refugee employment and integration in the Netherlands (de Vroome & van Tubergen, 2010).

#### The Purpose of the Training: Stimulating Agency

**Fig. 1:**
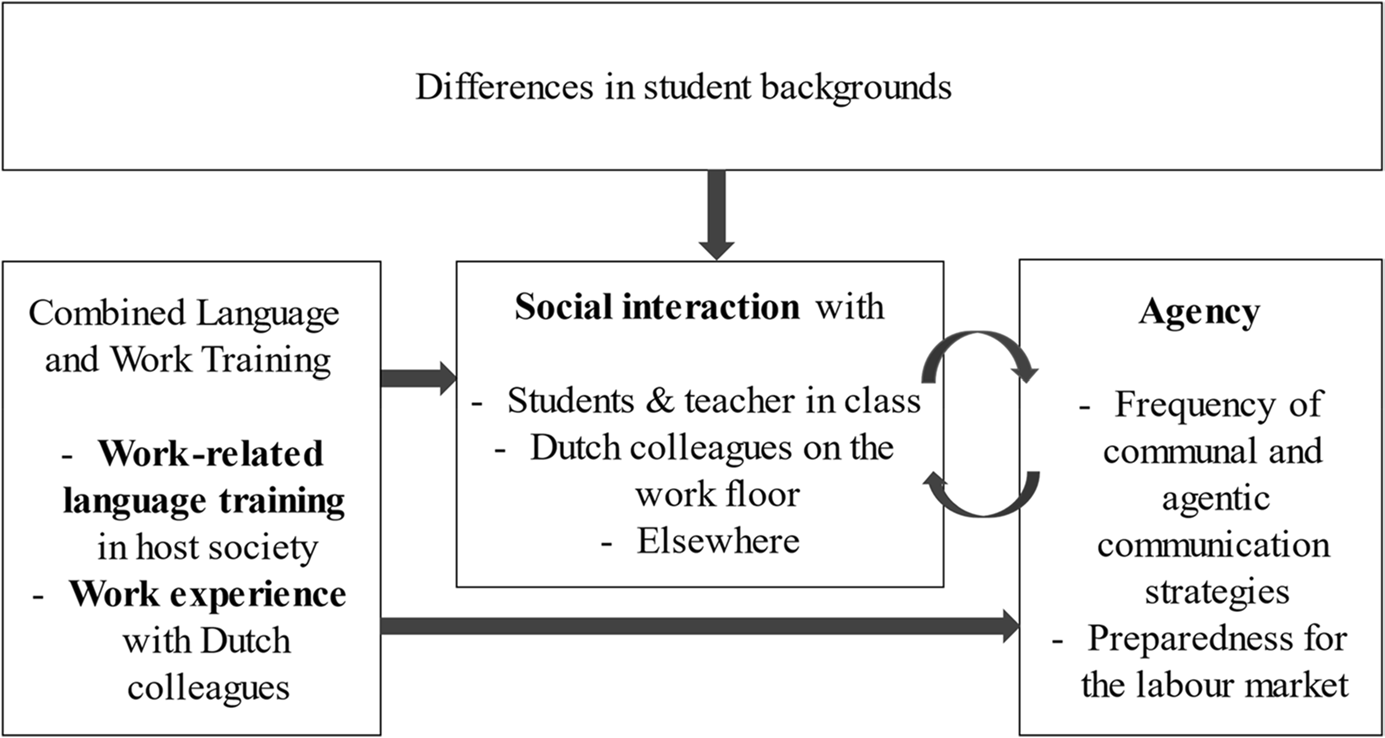
Conceptual model of the training. *Note.* The conceptual model of the training as derived from literature

As language proficiency and social contacts are important for refugees’ economic and societal integration, governments and non-profits increasingly develop integration programs combining these aspects with the goal of promoting early self-sufficiency and sustainable integration. These trainings are thought to promote agency, which is defined as an individual’s capacity to act (Samman & Santos, 2009). They aim to support a sense of ownership and control through creating an environment wherein own skills can be developed and strengthened in the new cultural context. Learners within these programs are expected to increasingly take charge of their own learning process (learner agency; Larsen-Freeman, 2019) to further strengthen their skill development, increase confidence in their own capabilities, and ultimately support labour market preparation (see conceptual model, Fig. 1).

Agency can manifest itself in different ways. This study argues that agency may show in two ways: (1) via language use, such as the frequency of interactions and the type of language strategies used; (2) via better preparedness for the labour market. The next section elaborates on why and how we investigated these aspects of agency.

##### Agency and Communication Strategies

We study language as an interpersonal process during which individuals can express themselves. *Language use*, one aspect of language proficiency, specifies with whom, where, and how one communicates, and is therefore relevant in immigrants’ integration processes (van Tubergen & Kalmijn, 2008). Considering the traditionally slow learning pace and diverse language use of adult literacy learners (Kurvers, 2015), we apply a process-oriented, micro-level analysis of language use in this research.

We focus on language use in the classroom in terms of *communication strategies*, taking into account social interactions and exploring agentic behaviour within language use. Based on interpersonal theory (e.g. den Brok et al., 2004; Pennings et al., 2018), we differentiated two dimensions of communication in the classroom: agency and communion. While agency describes how an individual engages with others (i.e. self-direction), communion reflects how an individual communicates with others (i.e. focussed on relational communication). Language use is thus characterized by differing levels of both agency and communion. Since language learning is a dynamic, relational, and temporally situated process (Larsen-Freeman, 2019), our focus on interactions and agency provides opportunities to explore individual language development in more depth while acknowledging the context in which communication occurs.

Communication strategies involve strategies to resolve barriers in interactions and/or difficulties in expressing oneself. They can range from basic strategies (e.g. asking for clarification) to more complex ones (e.g. joking, expressing an opinion; Ugla et al., 2019). For example, humour as a communication strategy is strongly related to the idea of language play (Davies, 2015). While joking can further contribute to the development of language skills, it necessitates a certain level of proficiency in the target language (Bell, 2005) and thus reflects a higher skill level. We define high-order communication strategies as those characterized by high levels of both agency and communion, as well as those requiring higher proficiency. We distinguish these from lower-level communication strategies, characterized by low levels of agency and communion. We expected that the level of communion and agency in communication of low-literate refugees will be higher after having participated in the program.

##### Agency and Preparedness for the Labour Market

The combination of work and language training furthermore could translate into better labour market preparation via agentic behaviour. As both language and literacy form the means to negotiate and access power in everyday social interactions (Tadayon & Khodi, 2017), they are fundamentally connected to an individual’s capacity to act. Especially in the resettlement context, language proficiency and literacy strongly link to identity construction and feelings of belonging in the new host society (Huizinga & van Hoven, 2018), and can support feelings of agency and self-esteem (Klenk, 2017). For linguistically isolated refugees, being unable to speak the language of the host society can impact feelings of self-worth (Nawyn et al., 2012), whereas refugees proficient in the host country language express higher levels of autonomy, personal aspirations, and a sense of achievement (Salvo & de C Williams, 2017). As a place for personal growth, work experience can stimulate self-efficacy and self-esteem and positively impact how workers perceive their own capabilities (Castañeda et al., 2019). Similarly, systemic and cultural knowledge of the workplace can increase feelings of self-efficacy (Tip et al., 2020).

### The Current Study

The current study was conducted in the Netherlands. Of the cohort that received their asylum status in 2014 in the Netherlands, only 38% had found employment 4.5 years after their arrival, 75% working in temporary and 88% in part-time jobs, while 50% were dependent on welfare benefits (CBS, 2020). We longitudinally followed a training that combined language and work experience for refugees with low-literacy levels. Employing a mixed-methods design, we examine how the interplay between language use and contact relates to refugee students’ sense of agency. While we describe the outcomes of the training on a group level, we also examine who and why benefitted most through case comparisons. Overall, being able to communicate in Dutch and local work experience are expected to be related to an individual’s capacities to participate in the host society.

## Methods

### Research Collaboration

**Fig. 2:**
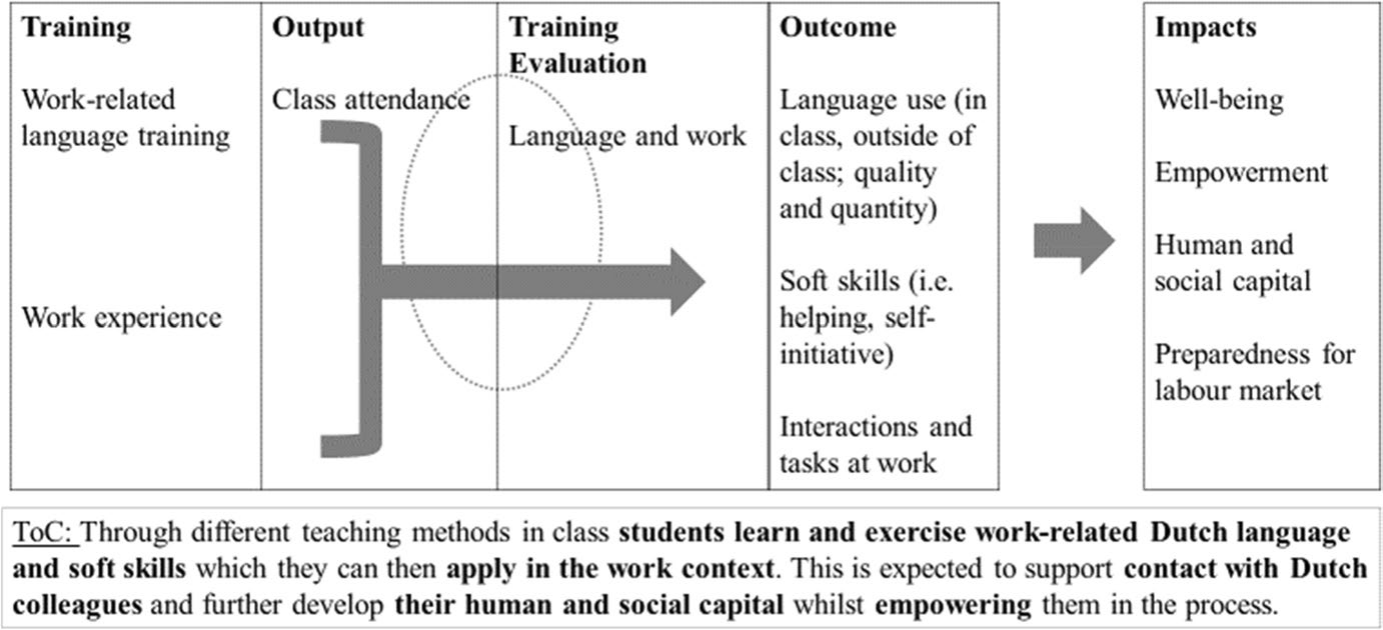
Theory of change of the training program. *Note*. Visualization of the theory of change as assessed through discussion with the program coordinators and teacher interviews at *t*_0_

We approached a Dutch regional training centre in the city of Groningen. We developed a mixed-methods evaluation of one of their programs together with school management and teachers. This centre collaborated with the local municipality and offered a combined language and work experience training program for low-literate refugees. First, we jointly constructed a Theory of Change (White, 2009), which is a description of how and why a specific intervention is expected to bring about a desired change in outcomes (see Fig. 2). Contrary to our conceptual model (Fig. 1), which was primarily based on existing research findings, the Theory of Change summarizes the program coordinators’ ideas behind specific training activities. This later enabled us to evaluate the implementation of the program. The first author regularly met with the coordinator to critically reflect about the research process. The theoretical and methodological approach was the responsibility of the researchers. Unfortunately, the COVID-19 pandemic prevented us to follow up on students’ work placements after the program.

### Training Description

The training lasted 1 year (January 2019–December 2019) and offered language training, cultural knowledge, and work experience. It included a morning program (4 days per week) consisting of language training, cultural knowledge training, and an afternoon work program 5 days a week.

The *language training* consisted of exercises related to listening, speaking, writing, and reading, including individual and group exercises. This included work-centred vocabulary (e.g. tasks, equipment, small talk), language coaching at work (once per week), the use of a digital training program developed for fostering literacy among adult refugees (Diglin), and a weekly workbook including personal reflections and learning goals. All refugees should reach the CEFR^1^ levels A1 and A2 at the end of the course. Two language teachers taught the group and were often supported by volunteers. Teachers and two work floor coordinators met regularly to discuss individual progress, issues, and work-relevant vocabulary.

The morning program also aimed to increase students’ *cultural knowledge*. Students were encouraged to ask questions about Dutch customs, share their own traditions, and compare them (e.g. Christmas, birthdays). Teachers used a weekly/monthly calendar as a structure to offer students a better understanding of Dutch society and to support students with their own scheduling (e.g. language tests, meetings with municipal coaches). Within the classroom, teachers and students reflected on the cultural appropriateness of certain behaviours and expressions in class and at work.

In the afternoon, students gained *work experience* in a second-hand shop (located in the same building), which functioned as a place of sheltered employment for people with a distance to the labour market. Students were engaged in different tasks, including sorting clothes and organizing the displays, working at the register, cleaning, picking up second-hand furniture, and working in the warehouse.

### Participants

The initial group consisted of nine students. After 4 months, one new member joined. Around six students were mostly present during the classes, two of them also working at an additional workplace. The group was diverse both in terms of age, ranging from early 20s to 40s, and cultural and linguistic backgrounds (Syria, Eritrea, Iran, Guinea, Afghanistan, and Nigeria). Eight students had at least one other person in class with whom they could speak their native language. We use the term low literates throughout to capture that students were either other literates (i.e. being able to write Arabic only) or low literates, expected not to pass the integration exams within the allotted three years (see Table 1).

**Table 1:** Overview of students

Participant	Progress in language use	Interactions in class, at work, and beyond	Motivation for the training	Previous work experience—match with tasks at work	Family	Language partner in class	Additional work
Motivated Mothers							
Helen	++	++	++	Yes, match	Married, mother of 3; partner in home country	Yes	No
Linda	++	++	++	Yes, no match	Mother of 2	No	No
Jol	++	++	++	Unknown	Mother of X	No	No
Struggling Speakers							
Nahla	--	--	-	No	2 adult sons in NL	Yes	No
Hakim	-	+ in class - outside class	+	Yes, no match	Married, partner in NL	Yes	No
Otherwise Employed							
Kidane	+?	++	-	No	Girlfriend, father to be	Yes	Yes
Omar	+?	++	-	No	Unknown	Yes	Yes
Rahim	+?	++	-	Unknown	Girlfriend in NL	Yes	Yes
Lonesome Learners							
Jesper	-?	-- outside class	++	Yes, no match	No	Yes	No
Yonas	+?	-- outside class	++	No	Uncle in NL	Yes	No

Participants are grouped according to their profile, in line with their progress in language use, social interactions in and outside of class, and their motivation. Additionally, participants matching with previous work experience, their family background, whether they have a language partner in class, and whether they have other employment is provided

### Procedure and Research Design

The Ethics Committee Psychology of the University of Groningen approved the study. Individual data was safeguarded through the non-sharing of individual-level data between researchers and the school. To ensure that all students would understand the research and its implications (Sudore et al., 2006), we first engaged in a trust building stage with the students (Shenton, 2004; the first author visited the group five times). The first author carefully explained the study and students’ rights with support of the language teacher. This information was re-iterated throughout the data collection before asking for verbal consent. Consent was implemented as a process in our design, rather than a one-time event, thereby ensuring continuous voluntary participation (Helgesson & Eriksson, 2011; Lidz et al., 1988). For all interviews, students were asked for permission to record the conversation. If permission was given, the interviews were recorded with a dictaphone. Otherwise, the first author took notes during the conversation and transcribed these after the data collection.

The students received snacks as compensation and were debriefed in an additional meeting. During this meeting, the first author presented the findings to the students and teachers in class and invited them to reflect and comment on the results to ensure the trustworthiness of the research (Shenton, 2004). Students agreed with the study findings. We derived recommendations for the program, which the authors presented to the school management.

**Fig. 3:**
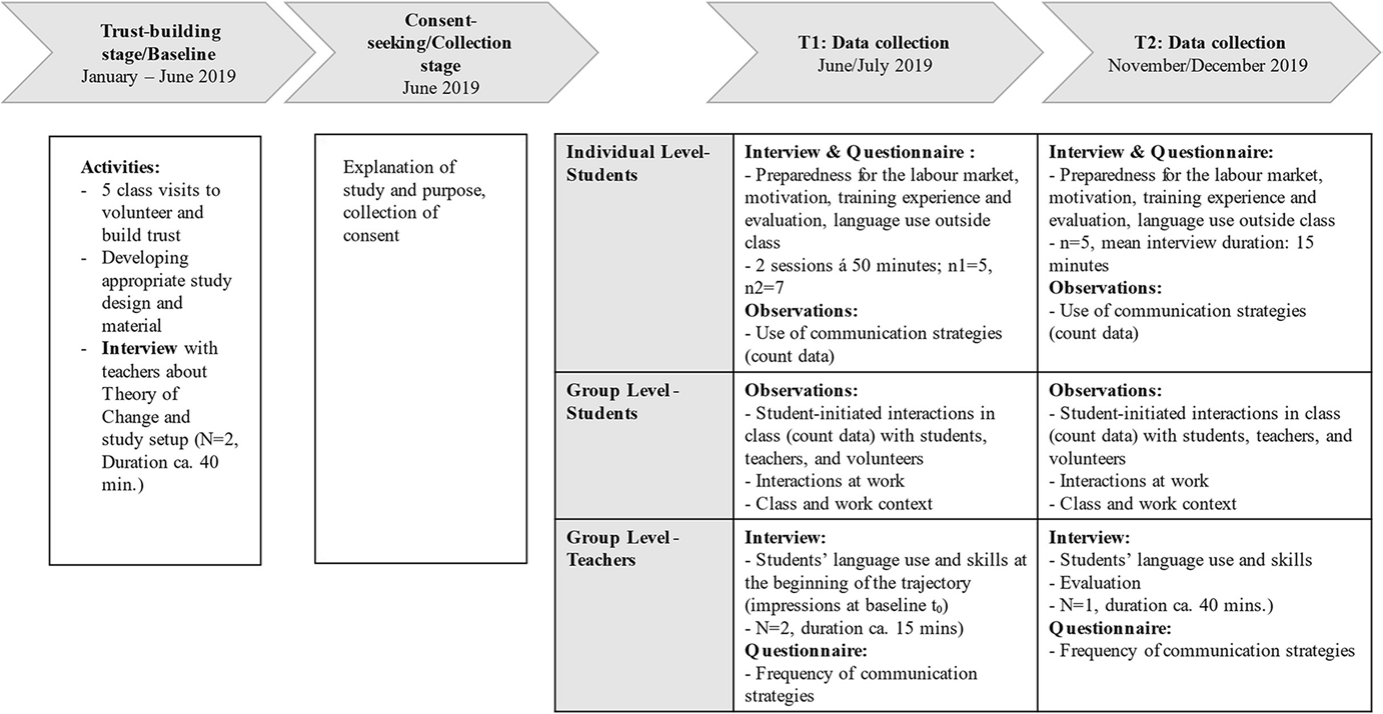
Study design

We employed a mixed-methods design and collected data through semi-structured interviews, observations, and questionnaires. All data was collected in Dutch for two reasons: Firstly, focusing on Dutch allowed us to study how and where students spoke Dutch. Secondly, the range of different mother tongues would have necessitated inviting multiple translators to the data collection process. However, we thought that this would disturb small group interactions, and potentially decrease trust towards the researcher. We employed a kind of 3 (time) × 3 (level of analysis) research design. We collected data at three different time points: Baseline, Time 1, and Time 2, and focussed on three different levels of analysis, namely (1) the individual level, (2) the group level, and (3) the teacher level, to gain a more detailed understanding of individual’s progress (for an overview, see Fig. 3). Due to the gradual consent procedure, we could not interview students at the baseline. During the first interview (time 1), a teacher was present to help with the communication in Dutch. In total, it lasted 120 min and was split up in two shorter meetings of 60 min. At time 2, the first author interviewed 3–4 students who spoke the same mother tongue together to ensure the understanding of questions and offer a safe environment. The second interviews lasted between 10 and 40 min (for a variable overview, see Fig. 3).

### Measures

#### Training Evaluation

Students evaluated the training twice (time 1, time 2) by answering two questions about the language class and the work experience on a 5-point Likert scale (“How do you like the class/work?”). We used a visual 5-point Smiley Likert Scale (Hall et al., 2016; Hansen et al., 2014) which was easy to understand ranging from 1 (very bad) to 5 (very good). We used the same scale to assess other student-rated items.

#### Agency

##### Communication Strategies

Two aspects of language use were assessed, namely language use inside and outside the classroom. The first author collected data of the communication strategies inside the classroom on all three levels through observations of students, interviews, as well as questionnaires.

Observations were used to track language use (type and frequency, based on Ugla et al., 2019) and interactions using a pre-set observation scheme. For each student in the classroom, we counted how often and with whom they initiated a conversation, and how often they used a specific communication strategy in the classroom (see Table 2).

**Table 2:** Overview of hierarchy of second-language (L2) communication strategies

Level	Communication strategy
Lower-order	Appeal for help (AH)
	Asking for confirmation (ACo)
	Asking for clarification (ACl)
	Ignorance acknowledgement (IA)
Higher-order	Expressing an opinion (O)
	Joking (J)
	Helping other (HO)
	Sharing (personal) information (SI)

Not in the categorization: language switch (LS). Language switch denotes students switching from Dutch to their native language

Teachers were interviewed twice to evaluate the group’s language use (baseline, time 2; 30–50 min), which was supplemented with a questionnaire on perceived in-class language use. At all measurement times, teachers reflected on the groups’ skill level and interactions between students.

##### Preparedness for the Labour Market

Data on preparedness for the labour market was collected on the individual level. We conducted semi-structured interviews with participants at time 1 and time 2 which were supplemented by a questionnaire. During the interview, students were asked about their training experiences, but also their aspirations.

The questionnaire for students included one item on perceived preparedness for the labour market: “I feel prepared to work”. To learn about how students would feel prepared, we assessed feelings of empowerment based on a Dutch questionnaire developed for social work (Depauw & Driessens, 2017). Specifically, the items assessed social attitude, positive attitude, and involved attitude. We chose four easily understandable items to assess empowerment: “I can share my experiences with others” (Involved Attitude), “I take satisfaction in things that are going well” (Social Attitude), “I know my strengths” and “I am generally optimistic about the future” (Positive Attitude). At time 1, total scale reliability can be considered adequate (*α* = .66, *N* = 6); however, scale reliability at time 2 was very low (*α* = .13, *N* = 5). Through the connection of the questionnaire to the interview, more in-depth discussions became possible.

At time 1 and time 2, teachers reflected on skill development at the group level during the interviews. This included information on punctuality, attendance, self-initiative, and soft skills like helping behaviour.

## Results

We first present our group-level findings and next offer individual profiles to gain deeper insights into the interplay between language use and intergroup contact, in line with our theoretical framework.

### Training Evaluation

Both class (*M* = 5 both measurements) and work (time 1 (*N* = 7): *M* = 4.14, *SD* = 1.46; time 2 (*N* = 5): *M* = 4.2, *SD* = 1.79) were evaluated very positively. Important to note, students’ reactions were more critical about the work experience, which we discuss below.

### Agency

#### Language Use

We expected that students show more communal and agentic communication after participating in the program. Next to combining the counts of higher-order and lower-order strategies, we analyzed the differences for each strategy individually. On a descriptive level, students used slightly more communication strategies over time.

Additionally, we assessed teachers’ impressions of in-class communication strategies at time 1 and time 2 (see Table 3). Teachers perceived an increase in all strategies. Only language switch decreased over time (*d* = −2). In the interview at time 2, the teacher made a general distinction between who made progress and who did not. The latter occurred mainly among individuals who entered the program with lower levels of literacy. Though language use improved also for this group, the teachers commented that “the steps are so small that they cannot be used for their daily functioning”. Teachers pointed out difficulties in promoting learner agency, as students were focused on doing well on exercises and tests (e.g. looking at the answers of other students).

**Table 3:** Descriptives of class observations and teacher evaluations of communication strategies

		LS	Lower-order strategies					Higher-order strategies					Total
			AH	ACo	ACl	IA	Overall	O	J	HO	SI	Overall	
Class observations	Counts *time 1*	6	3	1	8	0	12	10	6	5	12	33	45
	Counts *time 2*	5	1	1	12	2	16	4	15	9	8	36	52
Teacher evaluations	Mean *time 1*	4.0	2.5	3.5	2.5	2.0	2.62 *SD*=.18	2.0	2.5	3.5	2.5	2.63 *SD*=.88	
	Mean *time 2*	4.0	4.0	4.0	4.0	4.0	4	3.0	4.0	5.0	4.0	4.0	

Class observations represent frequencies of observed communication strategies at the group level. Teacher evaluations represent rated frequency of communication strategies on a 5-point Likert scale. At time 1, both teachers filled in the questionnaire; at time 2, one teacher was present. At time 1, 7 students were present during class observations. At time 2, 6 students were present

**Fig. 4:**
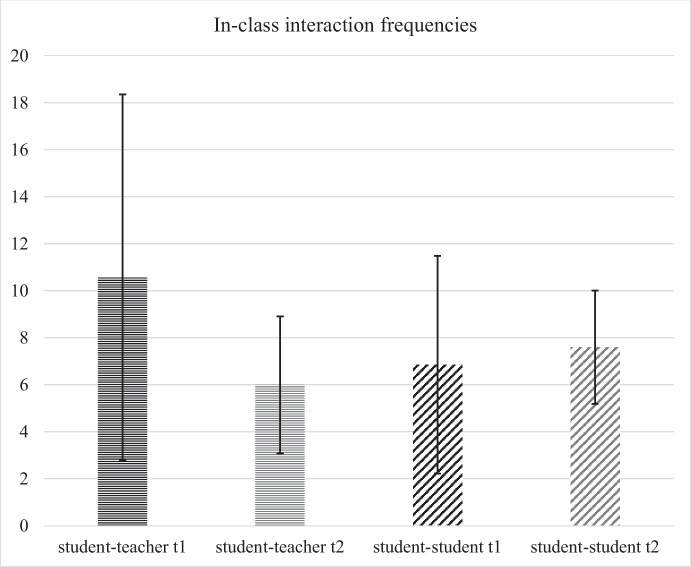
Bar graph of student-initiated interactions at time 1 at time 2. *Note.* Group level in-class interaction frequencies between students and teachers as well as students and students at time 1 and time 2. In contrast to the communication strategies, the interaction frequencies also portray interactions not captured by the categorization. *N* at time 1= 7, *n* at time 2= 6

Overall, our data suggest that students increased their use of communication strategies, and used language switching less frequently. Interestingly, at time 2, mainly students who still had a very low level of Dutch and their respective language partners used language switching to understand questions and/or explain tasks to their fellow students. Despite teachers reporting slow progress, our observations reflect that students used communication strategies more frequently, and that differences in interaction frequencies at the group level became smaller (see Fig. 4). Furthermore, interaction frequencies show that students interacted more with other students rather than with the teacher, indicating both more agentic and communal communication.

#### Preparedness for the Labour Market and Intergroup Contact

We expected that students would feel better prepared for the labour market through the combination of language, intergroup contact, and work. Our survey data show high preparedness at both measurement points (see Table 4). The interviews offer insights why the perceived preparedness was so highly rated, as students aimed to indicate their strong motivation to work. Their impressions of preparedness were based on the tasks they engaged in at work and in the training. Most students remarked that they already possessed the skills needed at work (e.g. cleaning) and that they therefore felt prepared, except for their Dutch skills. Two students critiqued the work tasks and the mismatch with their own aspirations, implying that they did not feel prepared for the work they hoped to find in the future. Another student stated a lack of meaningful contact with his Dutch colleagues. According to him, the vocabulary used at work was restricted to daily tasks: “They don’t talk with us, they only look for work for us”, to a point at which, at the end of the day, these words were the only things stuck in his head (“Clean up, destroy, clean up, destroy, clean up, destroy”). Based on observations and interviews, it became apparent that the intended intergroup contact in the training rarely happened. For example, the storage workspace, which also functioned as a break room, created space for interaction, but not necessarily positive ones. Related to the idea of power imbalance in communication (Nawyn et al., 2012), Dutch colleagues often appeared to joke about the students needing to work harder while taking a break themselves, which was met with frustration by the students.

**Table 4:** Descriptives of student questionnaire on preparedness for the labour market

Item		M	SD
I feel prepared to work	Time 1	4.17	.41
	Time 2	4.80	.45
Involved attitude	Time 1	4.67	.52
	Time 2	3.80	1.10
Social attitude	Time 1	4.33	1.63
	Time 2	4.20	.84
Positive attitude	Time 1	4.25	.82
	Time 2	4.60	.42

*N* at time 1 = 6, *N* at time 2 = 5

To learn more about how students felt prepared, we asked questions related to empowerment (see Table 4). In combination with the interviews, the items relating to evaluation of own strengths and connection with others prompted more in-depth discussions. At time 2, Yonas, Hakim, and Rahim were interviewed together. In this constellation, Rahim took on the role of a translator for Hakim but also provided examples from his own life, which prompted the others to further elaborate on the questions. While Hakim mainly struggled with the language, Yonas had troubles filling in the questionnaire. Despite thinking about his strengths and encouraging fellow students, Yonas was unable to formulate a strength, which seemed to frustrate him. Rahim passionately elaborated on his football skills (“I am the best!”) and supported Hakim in expressing his piano skills. Additionally, the question on being able to share experiences with others resulted in additional conversations about loneliness and adjustment difficulties, which is also reflected in the quantitative data. Yonas shared that he is often on his own, watching TV, and that he does not have social connections that he could turn to. This was followed by Rahim expressing that this is difficult for him, but also for the rest of the group, because they are often alone. He stated that, even though all students are close, everyone has their own issues and that those are too overwhelming to be able to help each other. Despite Hakim’s low level of language proficiency, he also followed the conversation and agreed with the others (“difficult”).

The teacher’s impression corroborated this mixed finding: Some students showed more self-confidence in class but this varied greatly, with some remaining dependent on encouragement of the teachers. They also brought up the negative role of shame and comparison with other refugees who achieved higher levels of proficiency in shorter time, with students often feeling frustrated with their own progress. At work, some were very conscientious and showed self-initiative, while others thought of the work experience primarily in terms of volunteer work into which they do not need to invest much effort. Similarly, some students remained dependent on continuous task instruction and supervision at work. Still, students often helped each other in and outside of class.

### Differential Training Impacts: the Role of Motivation and Social Contacts

In addition to the group-level outcomes, we analyzed individual-level data for each participant. This analysis confirmed that the training differentially affects students, with some students making high or moderate progress and others less, considering interaction frequencies and the range of communication strategies they use. Therefore, it is crucial to examine for whom and why this program was effective (Walton & Yeager, 2020). Gaining a better understanding of the underlying processes can, firstly, inform our understanding of the interplay of language use and contact during the integration procedure, and, secondly, promote the long-term impact of such trainings.

We synthesized the data across the three time points to identify how these differing outcomes might be related to characteristics of the students or the training itself. In doing so, the impact of the training most prominently related to differing degrees of students’ motivation as well as social activity in class, at the workplace and beyond. In the following, we describe the language use and agency in four distinct student profiles, characterized by their motivation and social activity.

### Profile 1: Motivated Mothers—Jol, Helen, and Linda

**Fig. 5:**
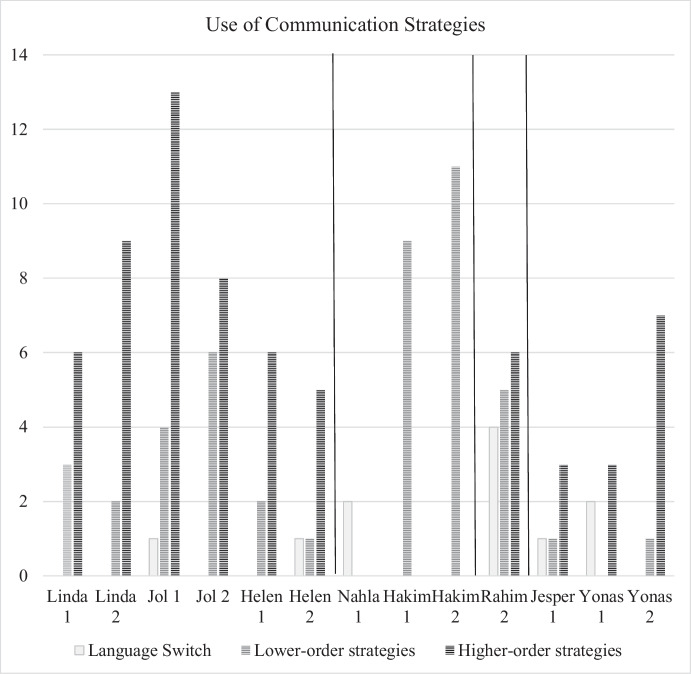
Bar graph of students’ communication strategies per profile at time 1 and time 2. *Note.* Students are grouped according to their profiles, namely Motivated Mothers, Struggling Speakers, Otherwise Employed, and Lonesome Learners. Frequencies represent use of language switching, lower-order and higher-order communication strategies at time 1 and time 2. *N* at time 1 = 7, *N* at time 2 = 6

**Fig. 6:**
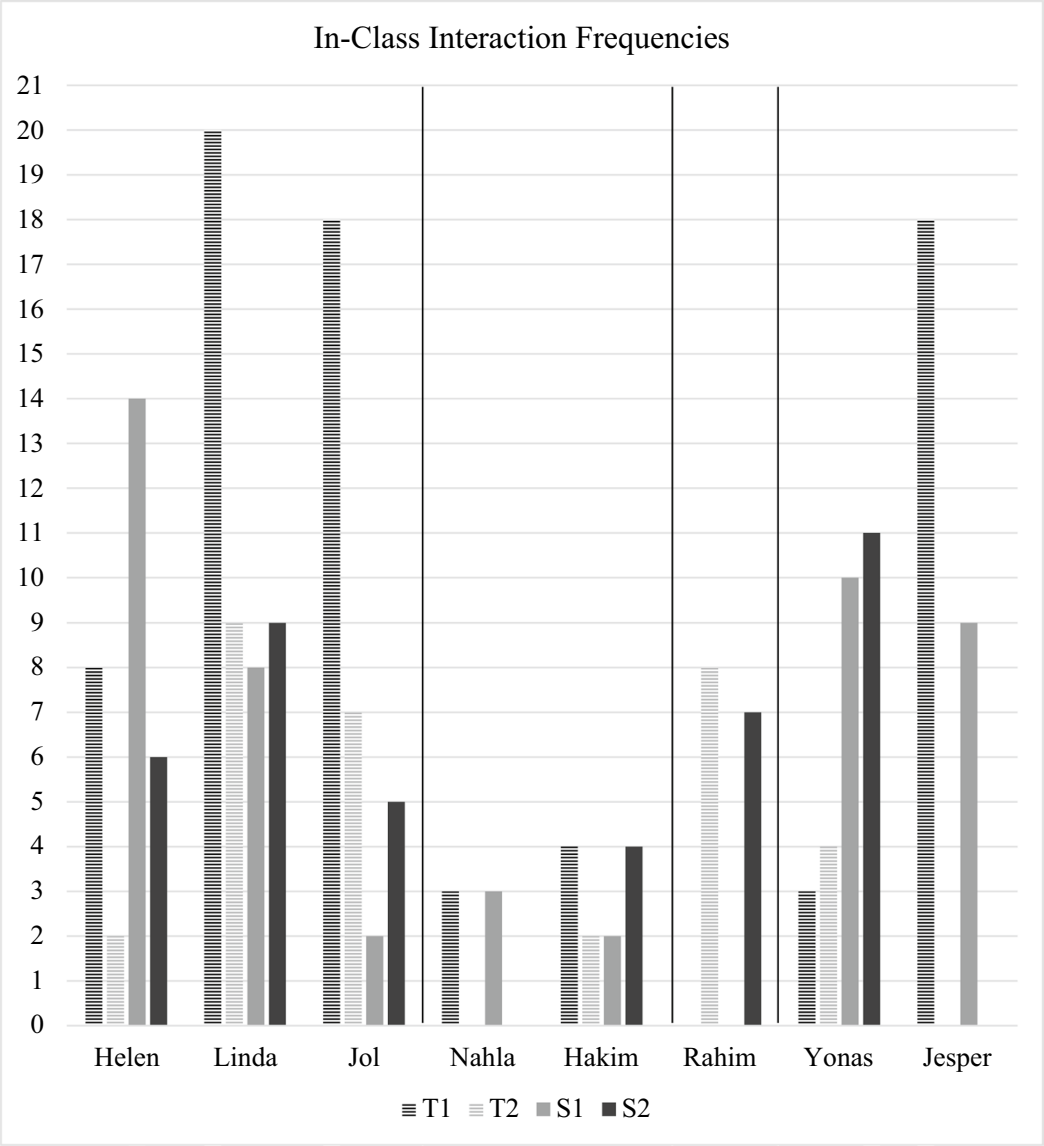
Bar graph of student-initiated in-class interactions per profile at time 1 and time 2. *Note.* Students are grouped according to their profiles, namely Motivated Mothers, Struggling Speakers, Otherwise Employed, and Lonesome Learners. Frequencies represent interactions with other students and teachers at time 1 and time 2. *N* at time 1 = 7, *N* at time 2 = 6

In the literature, female refugees are often thought to face additional barriers to employment, as reflected in low labour market participation. This has been explained by less contact with natives, lower levels of language proficiency, and cultural norms (Klenk, 2017). In the current study, a group of women with young children and no partner in the Netherlands gave a different picture. This group included Linda, Helen, and Jol. Of all students, they were among the most active in class when it came to language use, but also with regard to the diversity of communication strategies they employed (see Fig. 5) and the amount of interactions in class (see Fig. 6). Their in-class behaviour reflected their communication strategies: They had a central role in the group, often offering support or helping others with study material or exercises, as well as using higher-order strategies of joking and expressing opinions.

At the same time, they also had a strong sense of personal agency. Jol was always clear in what she wanted to know and asked clarifying teacher-directed questions focused on understanding. In the beginning of the research, she even asked whether she could get a reference letter from the primary researcher. This is resonated also in Linda’s statement about her motivation: “I try my best for school. It’s not easy for me, but I try. Every time I come I talk to colleagues, to friends. Me, it is good for me. Communication with the teacher, with my friends”. Helen also remarked that she talks Dutch at home with her children.

Their sense of agency is also evident from the data on preparedness for the labour market and reflection on work. While they shared some difficulties in terms of intergroup contact and tasks, they all saw the usefulness of both the classes and the work, even in light of their child-rearing responsibilities. All of them had childcare responsibilities while taking part in the trajectory, which may have provided additional opportunities for contact with Dutch people, for example during school visits, but potentially also in terms of motivation.

#### Profile 2: Struggling Speakers—Nahla and Hakim

Profile two consists of Nahla and Hakim; both had family ties in the Netherlands. They did not communicate much throughout the trajectory, with both asking students who share the same native language to explain or translate (see Fig. 5). Both seemed to struggle with understanding and expressing themselves, as reflected in few in-class interactions (Fig. 6). While Hakim was more open in conversations through translations by fellow students, Nahla was not.

As for work motivation, Nahla was often approached for taking too many breaks at work, eventually leading to her not being allowed to continue her internship at the second-hand store in December. She hardly interacted at work during observation moments, which suggests that she did not have many opportunities for speaking Dutch. Her Dutch proficiency remaining at such a low level prevented communication with colleagues. In contrast, Hakim asked many clarifying questions during work, to ensure he was doing his tasks properly. However, most of these interactions were with other students rather than natives, and were restricted to a limited vocabulary. Nahla and Hakim were also the oldest students in the group. With age often being negatively related to L2 learning pace, this could be one explanation for their lack of progress. Still, throughout the trajectory, both Nahla and Hakim grew more comfortable interacting with others in the group.

#### Profile 3: The Otherwise Employed—Rahim, Kidane, and Omar

Profile three consists of Rahim, Kidane, and Omar, who all had additional work, and for whom attendance was problematic. Next to their jobs at an Arabic supermarket, in food delivery, and in bicycle repair, they worked at the second-hand store, albeit in a limited capacity. Having more varying work experience, it became clear they preferred working at their other jobs. Lack of communication with Dutch colleagues and task preferences were key reasons for this. As Omar put it: “This work is not for me. It is not my work”. Nevertheless, all three expressed appreciation for the supervisors’ support at the second-hand store. Rahim also mentioned that the other job had more opportunities for him to speak Dutch. Although their attendance was irregular, this group was among the most talkative of the class (see Figs. 5 and 6), which could be due to the additional Dutch contacts they had outside of class, both at work and in their private life.

#### Profile 4: Lonesome Learners—Yonas and Jesper

The final profile includes Yonas and Jesper and is characterized by a lack of social connections. Despite Yonas being one of the more advanced students in the class, his in-class communication remained limited (see Figs. 5 and 6). Still, he attended regularly and was described as a hard worker who would perform well on the labour market. His motivation to attend was related to being able to speak his first language in the class context (i.e. with Helen). His primary goal for his future was to get paid work as soon as possible, no matter what (“just work”). Jesper was similarly motivated to get a paid position as soon as possible, to become independent from social welfare benefits. Even though Jesper often initiated conversations with others outside the class context, he was frustrated with his lack of social connections, especially with Dutch people. While they both mentioned to regularly speak Dutch with their neighbours, they expressed their loneliness and inability to share their experiences with others. Neither of them had family ties in the Netherlands.

## Discussion

This research examined how a combined language and work experience training for refugees with low-literacy levels influenced their agency in terms of language use and preparedness for the labour market. While combined programs have been suggested to promote the labour market integration of refugees, very little is known about their effectiveness, especially so for programs targeted at low-literate refugees. Based on Ager and Strang’s integration framework (2008), we elucidated how and why this approach may theoretically promote integration via intergroup contact. We used a mixed-methods approach to follow one such program, and examined the group-level outcomes of agency in terms of communication strategies and preparedness for the Dutch labour market. Due to the high variability in outcomes, we described cases to illustrate how students’ motivation and social connectedness can explain the differential training impact.

### Findings and Interpretation of Results

We expected more frequent communal and agentic communication. Both the observational count data and teacher observations indicate that the frequency of communication strategy use increased during the training, whereas the incidence of language switching decreased. Results suggest that students developed more agency with respect to frequency of communication strategies, as students interacted and communicated more in general. Students also developed stronger communal communication towards each other (Pennings et al., 2018), and their dependence on the teacher decreased. Even though individual differences in language use and progress were prevalent throughout the trajectory, the interaction frequencies also indicate fewer differences at the group level at time 2. Despite the slow functional progress in language proficiency, our analysis does indicate a development towards more and higher-order communication.

We also expected increased feelings of being prepared for the Dutch labour market because of the training. Feeling prepared for the labour market was rated highly throughout the training; also due to work tasks that students already felt prepared to do. The high self-rating of preparedness also reflected students’ strong motivation to work. Investigating how students felt prepared, it became apparent that some struggled with realizing their own strengths. Additionally, loneliness and lack of meaningful contact, both within and outside the program, negatively affected their well-being. As well-being and intergroup contact are positively connected to labour market outcomes, these factors—partly outside of the control of the program—may reflect lower labour market preparedness.

While students may have increased agency in terms of language use, this did not necessarily translate into being able to experience mastery and control in the work context. Considering students’ experiences, this was related to a mismatch of work tasks with their own aspirations, as well as the lack of meaningful intergroup contact at work. Even though the program set out to promote self-sufficiency, it left little room to consider refugees’ own agency. Nevertheless, some students showed high levels of agentic behaviour, both at work and in terms of language use. We therefore compared individual cases to have a closer look at influential factors.

Through the grouping of profiles and their comparison, we noticed the following patterns. Firstly, and unexpectedly, the group of the motivated mothers made most progress in terms of host language use, going against some findings in the literature, which put refugee women at a disadvantage in the integration procedure. Reasons for their success in the training could be related to their motivation (caring for their children) as well as access to linking social capital they may have had through their children. However, it also became clear that a good compatibility of the combined approach with child-rearing responsibilities was crucial and needs to be taken into account. Relatedly, the group of *Otherwise Employed* may have benefitted from more bridging contacts through other work placements. Through the other profiles, it became clear that students’ learning pace differed greatly, which could be partly explained by age. Furthermore, the negative impact of loneliness and lack of social capital on agency and well-being was evident, as well as the positive impact of intergroup contact inside and outside of class on language use.

The differential program impact can be explained by the following. Firstly, language development within second-language literacy courses is slow and is characterized by considerable heterogeneity in terms of progress (Kurvers, 2015). This can be related to a number of individual-level factors, such as age, contact with Dutch speakers, and attendance (Kurvers, 2015), which we could also recognize in our study trough the comparison of students’ profiles. Secondly, the success of the training depends on both the attendance of students, but also on the interaction with Dutch colleagues on the work floor. While students valued both the supervisors and the teachers, opportunities for (meaningful) contact with Dutch colleagues appeared to be very limited, thereby restricting the very mechanism through which the intervention of combining work and language training is supposed to be effective.

### Limitations

Our study has two main limitations. First, we tried our best together with the vocational training centre to develop a robust study design. This pilot program was only designed for a small group of students. Unfortunately, we could not find an appropriate comparison group of other newcomers with low-literacy levels for a matched comparison group. Some students were not regularly joining the training program so we were not only able to collect data of all at each time point. Due to these reality constraints and the small sample size, we cannot draw any quantitative causal conclusions. Notwithstanding the non-generalisability of the results, the mixed-methods approach enabled us consider the context in which interactions occurred and the focus on within-group variations and process offered new insights on who profited most from the program. Especially in light of scarce research with low-literate refugees, we hope that the present research can contribute to more tailored and effective integration policies We are confident that the reported results are stimulated by the training participation and not participants’ natural language acquisition as a function of time spend in the Netherlands. All participants mentioned that this program offered a safe space where they could practice and improve their communication skills, which they could otherwise not have learned outside the classroom.

Secondly, we could not follow up with students to see whether they found a job and how quickly. Due to the COVID-19 pandemic, all schools had closed and meeting in person was impossible. We tried to stay in contact but were able to only briefly follow up with two students. We therefore cannot draw conclusions about the program’s effectiveness for promoting a quicker transition into the labour market. Despite constraints related to the data collection (e.g. language barriers, inexperience with questionnaires), the mixed-methods approach of this study allowed to explore the potential effect of such trainings for refugees with low literacy, a group that is often understudied. We hope that our approach provides guidance for future studies that aim to improve instruments for the quantitative monitoring of non-WEIRD populations, allowing them to better assess agency and empowerment in the resettlement context for understudied populations.

### Theory-Driven Practical and Policy Implications

Based on our findings, we propose two main practical implications for the program which form the basis of one main policy implication. Firstly, dual trajectories like the one under investigation consider both work and language as important for the development of self-reliance; however, focusing on self-efficacy instead might hold more potential. Work is thought to function as a space for experimental learning, and host country language skills, with their strong connection to literacy, are crucial for self-sufficiency in a new society (Blake et al., 2019). However, the assumption that the simple combination of work experience and language training will foster agency may be misleading, unless close attention is paid to the underlying mechanisms. Shifting the focus of these trainings to self-efficacy as an outcome might be more fruitful. Through focusing on self-efficacy, which is strongly related to positive well-being of refugees (Tip et al., 2020), these trainings could focus more on how opportunities for experiencing self-efficacy can be integrated in class and at work. Additionally, such a focus would shift from a “deficit perspective”—which emphasizes what refugees are lacking—towards a more positive psychology of refugee integration (Cobb et al., 2019). While human and social capital are crucial for the economic and social integration of refugees, it is imperative to take into account lived (integration) experiences and factors that positively contribute to refugee mental health and well-being (Cobb et al., 2019). Such a perspective goes in hand with a more situated view on learning and language use and shifts the responsibility for integration from the refugee to a shared responsibility of all involved actors. While language and employment, but also social connections and systemic knowledge, seem to be related to self-efficacy (Tip et al., 2020), opportunities for language use, meaningful social contacts, and opportunities to experience self-efficacy seem crucial to foster further skill development, promote well-being, and language skills. Including these as focal points of combined trainings could further promote societal integration and language acquisition.

Secondly, the specific target group of low-literate refugees needs to receive more attention to avoid the low-pay, limited language trap (Braddell & Miller, 2017). With limited Dutch skills, refugees may enter employment in which they have only limited contact with Dutch people. With the workplace as the most important and convenient location for interaction, language development, and language support, the development of individual learning strategies, goals, and realistic perspectives for work should be supported as much as possible within these integrated approaches, which necessitates opportunities for meaningful intergroup contact. Our findings gain particular relevance in light of the revised Dutch integration law (2022). This law aims to promote societal participation, and programs which stimulate language learning and work experience at the same time are currently being implemented, though rarely evaluated.

To sum up, our practical implications stress the importance of facilitating meaningful intergroup contact at work. While we consider the development towards combined integration trajectories positive, the premise that these new programs in itself lead to intergroup contact is insufficient to promote the intended faster integration of refugees. Instead, programs need to consider the opportunities for meaningful intergroup contact, especially since social connections promote integration at the local level, but also stimulate participation in other domains (Ager & Strang, 2008). Theoretically, meaningful intergroup contact may be facilitated through feelings of mutual respect, shared values, trust, and self-disclosure (Ager & Strang, 2008; Vezzali & Stathi, 2017). We propose that close attention is paid to these mechanisms when developing combined programs, and that these programs should consider the individual goals of participants to promote feelings of self-efficacy and belonging.

## Conclusions

The present research offers new important insights into the set-up of programs directed at increasing the societal participation and self-sufficiency of refugees with low levels of literacy. Based on our results, we highlight the need for such programs to structurally implement, firstly, opportunities for contact with Dutch colleagues and, secondly, opportunities for experiencing self-efficacy at work. Combined work and language trainings should not simply assume their effectiveness but rather critically evaluate the opportunities for language use and self-efficacy they provide. Future research should further develop appropriate methodology to evaluate the effectiveness of these approaches and should move towards a positive psychology of refugee integration by, e.g. assessing self-efficacy, belonging, and well-being. Additionally, more research is needed to examine the interplay between language use, social contacts, and employment for refugees, especially those with low literacy. Finally, we encourage more qualitative research exploring what meaningful contact constitutes for newcomers.

## Supplementary Information

Below is the link to the electronic supplementary material.

Supplementary file1 (DOCX 25.5 KB)

## Data Availability

The data that support the findings of this study are available on request from the corresponding author. The data are not publicly available due to privacy or ethical restrictions.

## Footnotes

1 Common European Framework of Reference for Languages
