# Supplementary material for: Combining Language Training and Work Experience for Refugees with Low-Literacy Levels: a Mixed-Methods Case Study
Source: J Int Migr Integr. 2023 Mar 25:1–27. Online ahead of print. doi: 10.1007/s12134-023-01028-6 (PMC10039336; doi:10.1007/s12134-023-01028-6)
Supplement: Supplementary file 1 — Supplementary file1 (DOCX 25.5 KB) [file 12134_2023_1028_MOESM1_ESM.docx]

**Combining Language Training and Work Experience for Refugees with Low-Literacy Levels: A Mixed-Methods Case Study**

**Supplementary Information**

**Summary**

1. Description of the Dutch Integration System
2. Description of the Dutch Integration System

By acquiring a temporary residence permit, refugees are allowed to stay in the Netherlands for 5 years. In the Dutch integration system up until 2021, passing an integration exam testing language skills (at a minimum level of A2) and institutional knowledge is required for receiving a permanent residence permit. Newcomers are encouraged to pass the exam within 3 years. Should they not be able to pass the exam within the allotted time, they may be subject to additional fees (Engbersen et al., 2015; Bakker, Cheung, & Phillimore, 2016).

Although the Dutch integration law 2021 (in effect starting January 2022) plans to path the way for earlier labour market participation (NCOD, 2019; SCP, 2020), self-sufficiency was greatly expected from refugees in the integration procedure so far whilst simultaneously being hindered through the integration procedures (*agency paradox*; SER, 2019). For example, refugees were expected to choose and pay for their own civic integration and language courses from a personal loan of 10.000 Euros (i.e., municipalities are nore allowed to recommend schools in the free market; NCOD, 2019). With high governmental expectations of early self-sufficiency, especially low-literacy groups struggle with the amount of bureaucracy and lack of easily accessible and understandable information (Adami, 2009; Liempt & Staring, 2020). As a result of the self-sufficiency approach, only a small number of policies had been focused on these groups with a great distance to the labour market (Engbersen et al., 2015).

The change to the Dutch integration law, however, also aims to increase support for refugees with low literacy and low learning capacity through increasing self-sufficiency (i.e. knowing how to access services, find employment) and societal participation in the so-called z-route (zelfredzaamheidsroute; Rijksoverheid, 2018). Within this route, refugees who are not expected to pass the exams in the allotted timeframe might be eligible for an extension, which raises the deadline for passing the integration exams. This will be supported through switching from a sequential integration system (first language, then work), to a more simultaneous one (combining language and work training; Engerbsen et al., 2015).

Other changes to the integration law include a switch from a national to a municipal approach and support system for personal integration assistance. In effect, this entails that municipalities first have a broad intake session to then determine the appropriate integration route (1. B1 route: Dutch level B1 within 3 years, in combination with (volunteer)work, 2. Educational route: For younger refugees, focused on getting a school diploma, 3. Z-route: Route for those to which the other two are not realistic to achieve; Rijksoverheid, 2018).

References

Adami, H. (2008). *The role of  literacy in the acculturation process of migrants*. Council of Europe. <https://rm.coe.int/CoERMPublicCommonSearchServices/DisplayDCTMContent?documentId=09000016802fc1b7>

Bakker, L., Cheung, S. Y., & Phillimore, J. (2016). The Asylum-Integration Paradox: Comparing Asylum Support Systems and Refugee Integration in The Netherlands and the UK. *International Migration*, *54*(4), 118–132. <https://doi.org/10.1111/imig.12251>

Engbersen, G., Dagevos, J., Jennissen, R.,Bakker, L., & Leerkes, A. with the assistance of Klaver, J. and Odé, A. (2015). *No time to lose: from reception to integration of asylum migrants*, WRR-Policy Brief 4, The Hague: WRR.

Liempt, I., & Staring, R. (2020). *Nederland papierenland: Syrische statushouders en hun ervaringen met participatiebeleid in Nederland*. Sociaal en Cultureel Planbureau, Den Haag.

NCOD. (2019). *Inburgering 2021: Routekaart*. <https://www.ncod.nl/wp-content/uploads/2019/04/Routekaart-Veranderopgave-Inburgering-_versie-26-04-2019_.pdf>

Rijksoverheid. (2018, February 7). *Inburgering op de schop: Nieuwkomers zo snel mogelijk aan het werk, leenstelsel afgeschaft*. <https://www.rijksoverheid.nl/actueel/nieuws/2018/07/02/inburgering-op-de-schop-nieuwkomers-zo-snel-mogelijk-aan-het-werk-leenstelsel-afgeschaft>

SER. (2019). *Integratie door werk: Meer kansen op werk voor nieuwkomers.* <https://www.ser.nl/nl/Publicaties/integratie-door-werk>
